# Association of atrial fibrillation and clinical outcomes in adults with chronic kidney disease: A propensity score-matched analysis

**DOI:** 10.1371/journal.pone.0230189

**Published:** 2020-03-18

**Authors:** Chunxia Zhang, Jingli Gao, Yidan Guo, Aijun Xing, Pengpeng Ye, Yuntao Wu, Shouling Wu, Yang Luo

**Affiliations:** 1 Department of Nephrology, Beijing Shijitan Hospital, Capital Medical University, Beijing, China; 2 Department of Intensive Medicine, Kailuan General Hospital, Hebei United University, Tangshan, China; 3 Department of Cardiology, Kailuan General Hospital, Hebei United University, Tangshan, China; 4 Division of Injury Prevention and Mental Health, the National Center for Chronic and Non-communicable Disease Control and Prevention, Beijing, China; Kaohsiung Medical University Hospital, TAIWAN

## Abstract

**Objective:**

Atrial fibrillation (AF) is associated with adverse outcomes in the general population, but its impact on patients with chronic kidney disease (CKD) remains unclear. In this study, we assessed the association between AF and risks of all-cause mortality and stroke in Chinese adults with CKD.

**Methods:**

We enrolled adults aged 45 years or older with CKD (defined as an estimated glomerular filtration rate <60 mL/min per 1.73 m^2^ and/or proteinuria identified using the urine dipstick method) from the Kailuan study between 2008 and 2014. AF was identified by 12-lead electrocardiography or hospital discharge diagnostic codes. Mortality data were collected from the provincial vital statistics, and physician-diagnosed ischemic or hemorrhagic stroke was confirmed in the biennial interview.

**Results:**

Among the 21587 CKD adults, 216 patients were identified with AF, the median follow-up duration was 5.21 years (5.69 ± 1.96 years); During follow-up, there were 70 cases of death, and 16 cases of ischemic stroke and 6 cases of hemorrhagic stroke in the participants with AF in comparison with 2572 cases of death and 656 cases of ischemic stroke and 184 cases of hemorrhagic stroke among the participants without AF. After adjustment for potential confounders, AF was associated with an 86% increase in the rate of death (hazard ratio [HR], 1.86; 95% confidence interval [CI], 1.33–2.59, P<0.001), a 104% (HR, 2.04; 95% CI, 1.09–3.83, P = 0.026) and 325% (HR, 4.25; 95% CI, 1.74–10.36, P = 0.001) increase in the rate of ischemic stroke and hemorrhagic stroke, respectively. These associations were still consistent and strong after propensity score-matched analysis.

**Conclusion:**

Our study shows that AF is independently associated with increased risk of all-cause mortality, ischemic and hemorrhagic stroke in Chinese CKD adults. Future studies are required to elucidate the physiological mechanisms underlying this association.

## Introduction

It is estimated that the prevalence of atrial fibrillation (AF) in general Chinese adult ranges from 0.6% to 0.7% in 2010, that means about 8 million Chinese adults have AF at that time [1–4]. Meanwhile, chronic kidney disease (CKD) has become another important public health problem in China and its prevalence has reached 10.8% in 2010 [5]. With the ever-increasing burden of these two diseases, it seems important to understand the pathological role of AF and CKD towards the clinical adverse outcomes, especially when they are coexistence [6].

Currently, it is well established that AF and CKD are closely related diseases sharing some common risk factors and showing similar adverse outcomes. Studies have shown that AF is one of the independent risk factors towards adverse clinical outcomes like all-cause mortality and stroke in general population and end-stage renal disease (ESRD) patients [7]; AF also has a high incidence and prevalence among the patients with CKD, however, there is still limited data regarding to the potential effect of AF towards adverse outcomes in individuals with CKD [8]. As we mentioned above, there is about 100 million CKD population in China, it is necessary and urgent to make further exploration of this important issue among Chinese CKD individuals.

Therefore, we determine to fill this void by exploring the impact of AF towards adverse outcomes in a large community-based longitudinal cohort of Chinese adults with CKD enrolled in the Kailuan study. We hypothesized that AF would be associated with a higher risk for all-cause mortality and stroke in the population with CKD.

## Methods

### Study designs and settings

We conducted a population-based prospective cohort study using the health care data repository of the KAILUAN Health and Wellness Registry file (Registration number: ChiCTR-TNRC-11001489), which include 101510 participants starting from 2006. All participants were followed biennially to update information on the potential risk factors and newly diagnosed diseases. The research was performed on the basis of the guidelines from the Helsinki Declaration and was approved by the Ethics Committees of Kailuan General Hospital. The authors obtained written informed consent from the participants before they were enrolled in the study[9, 10].

### Study participants

**Fig 1:**
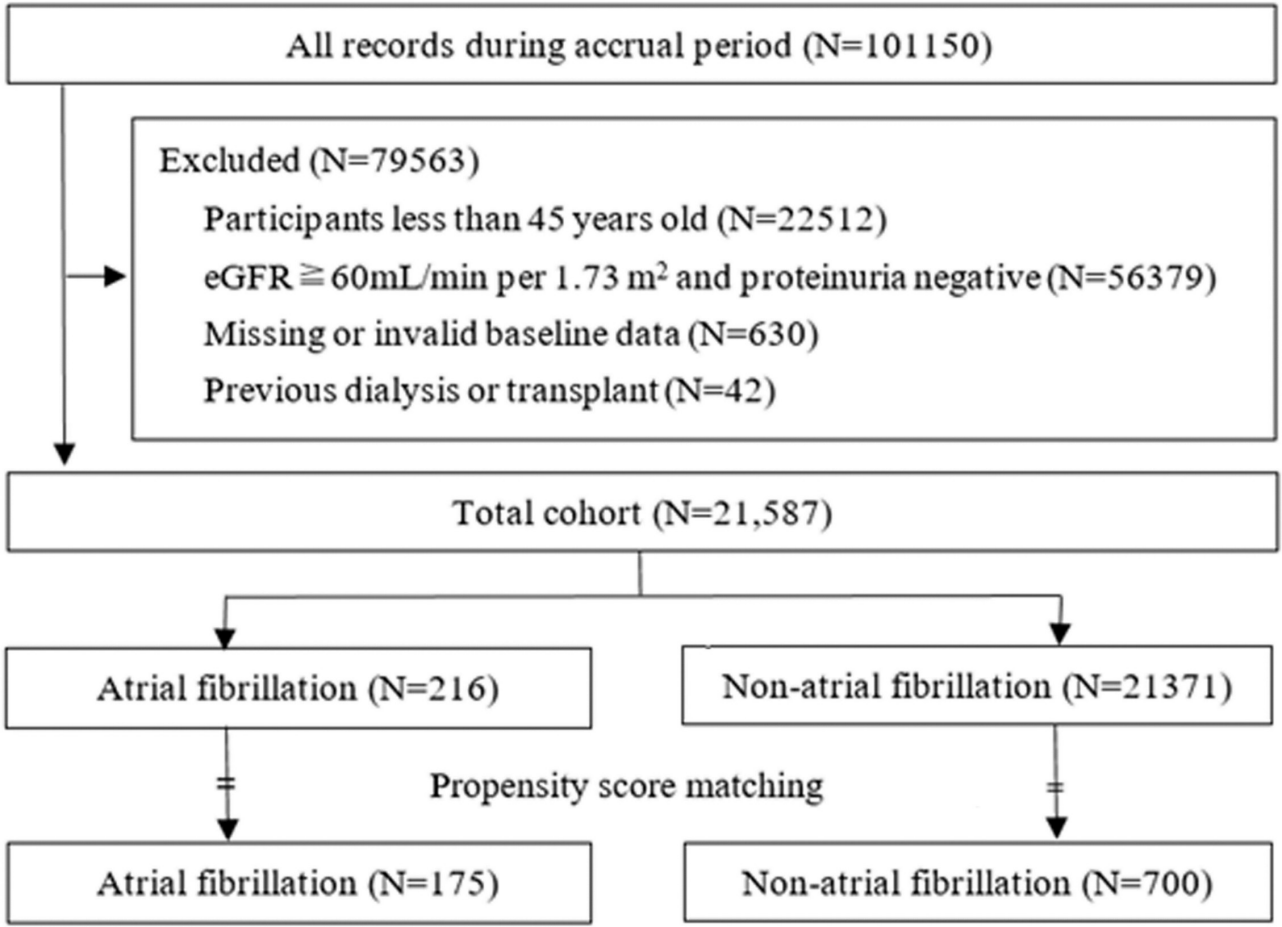
Flowchart of participant selection.

In the current analysis, we used the data repository of the KAILUAN Health and Wellness Registry file to create the study cohort. By the end of December 31, 2015, an initial 101510 participants were recruited in our study, the including criteria were: 1) 45 years old and above; 2) an estimated glomerular filtration rate (eGFR) <60mL/min per 1.73 m^2^, and/or whose proteinuria at entry was defined based on urine dipstick results and quantified as 1+, 2+, 3+, or 4+. The excluding criteria were: 1) participants with a history of kidney transplantation or dialysis therapies; 2) participants with missing or invalid baseline data, 3) participants with eGFR > 60 mL/min/1.73m^2^ and proteinuria negative. After these exclusions, a total of 21587 men and women 45 years and older with CKD remained in the analysis (Fig 1).

### Predictive variable and covariates

As the primary predictor, AF was diagnosed from cohort entry through December 31, 2014. AF was a time-updated exposure. Once a participant developed AF during follow-up, we contributed a person time to the AF exposure group, otherwise, we contributed time to the no AF exposure group before the diagnosis with AF.

Standard 12-lead ECGs were recorded in all participants by strictly standardized Procedures. The ECGs were read and analyzed using the Minnesota ECG classification at the Kailuan General Hospital reading center by electro-cardiographers [11]. The entry data were used as baseline data, participants provided socio-demographic characteristics and medical history information to a trained interviewer at each clinical examination. Anthropometric measures (e.g. height, weight, waistline, and standardized blood pressure measurements) were conducted. Hypertension was defined as systolic blood pressure (SBP) greater than 140 mmHg, diastolic blood pressure (DBP) greater than 90 mmHg, or self-reported use of antihypertensive medications. Diabetes was defined as fasting glucose greater than 126 mg/dl, random glucose greater than 200 mg/dl, or use of insulin or other anti-diabetic medication. Blood samples were obtained from all participants following standardized procedures. Serum creatinine and other blood assays such as total cholesterol (TC), LDL cholesterol, HDL cholesterol, triglycerides (TG), novel cardiovascular risk factors (C-reactive protein) were measured at the Kailuan General Hospital Laboratory [10]. The eGFR was calculated by using the Chronic Kidney Disease Epidemiology Collaboration (CKD-EPI) creatinine equation with an adjusted coefficient of 1.1 for the Asian population [12–14] and categorized as >90, 60 to <90, 30 to <60, and <30 mL/min/1.73 m^2^. Urine protein was defined based on urine dipstick results and quantified as none/trace, 1+, 2+, 3+, or 4+ [15]. For the multivariate models, blood pressure was included both using a categorical variable and as the systolic and diastolic blood pressure reading.

### Outcomes

The main outcomes included the all-cause mortality, ischemic stroke and hemorrhagic stroke events. Death information was collected from provincial vital statistics offices. Information on physician-diagnosed stroke events was collected in the biennial interview. To further identify potential stroke events, we also investigated discharge lists from the 11 hospitals. Different types of stroke were diagnosed according to the World Health Organization criteria, based on signs, symptoms, and brain imaging studies from computed tomography or magnetic resonance imaging [8]. Study participants were followed from the entry until the occurrence of the outcome events or until they were censored because of death, loss to follow-up, or the end of the study period on December 31, 2016.

### Statistical analyses

We used One-way ANOVA and the Chi-square tests to compare continuous and categorical variables, we also use the build-in command in Stata 14.2 to conduct the propensity-score match with demographic and clinical variables to adjust for potential confounding from imbalances in clinical characteristics between individuals with and without AF [16]. Demographic variables included age, sex, education level; clinical variables were smoke use, alcohol intake, hypertension, diabetes mellitus, waist circumference, SBP, DBP, TG, TC, LDL, CRP, and urine protein. Individuals with AF were matched in a 1:4 ratio with those without AF, and differences in clinical characteristics were determined, with P values > 0.1 considered well balanced. After all baseline characteristics were balanced in the propensity-matched model, AF exposure was the only independent variable in our Cox regression model.

We also generated Kaplan-Meier Curve, multivariable Cox proportional hazards regression relating AF to the outcomes of interest among pre- and post- propensity score-matched participants. In the multivariable Cox proportional hazards models, we repeated this analysis after adjusted for the set of covariates at baseline. All statistical tests were evaluated using two-tailed 95% confidence intervals (CI), and data analyses were performed using Stata statistical software version 14.2, and P < 0.05 was treated as statistically significant.

## Results

### Patient characteristics

Of the 21587 participants who met the inclusion and exclusion criteria of being aged 45 years or older with CKD, a total of 216 subjects (1.0%) developed ECG-detected AF at the entry of our cohort study. Then we were able to match 175 AF subjects to 700 Non-AF subjects by using the propensity score match (Fig 1). Baseline characteristics were compared between AF and Non-AF groups in pre- and post-propensity score-matched manner (Table 1). Before matching, participants with AF were older and more likely to have a history of hypertension, larger waistline circumference, and higher TG, TC, and LDL at baseline. After matching, the baseline covariates in the two groups were balanced with no statistically significant differences (Table 1).

**Table 1:** Baseline characteristics according to the presence of AF before and after propensity score matching.

Characteristic	Pre-Propensity Score Match			Post-Propensity Score Match		
	Non-AF	AF	P value	Non-AF	AF	P value^§^
**N**	21371	216		700	175	
**Demographics**						
** Age (y; mean ±SD)**	60.85±10.03	69.06±9.78	<0.001	68.76±10.26	69.00±9.93	0.771
** Male gender (%)**	59.94	65.19	0.055	88.86	86.29	0.342
** Education ≤9 years (%)**	86.40	89.19	0.270	89.06	90.57	0.672
**Clinical history**						
** Smoke use (%)**	36.27	30.58	0.091	29.43	28.57	0.824
** Alcohol intake (%)**	26.53	35.71	0.035	28.14	26.86	0.782
** Hypertension (%)**	23.22	29.29	0.044	30.29	28.57	0.660
** Diabetes (%)**	8.69	14.60	0.009	11.57	9.71	0.486
** Myocardial infarction (%)**	3.04	5.09	0.037	2.29	4.57	0.102
** Congestive heart failure (%)**	4.11	4.17	0.051	4.72	4.76	0.924
** Peripheral artery disease (%)**	2.45	3.37	0.040	3.77	3.30	0.705
** Prior strokes (%)**	3.32	5.56	0.022	5.19	5.66	0.657
**Waistline (cm; mean ±SD)**	88.06±9.95	91.51±11.33	<0.001	91.37±11.45	91.21±11.33	0.873
**SBP (mmHg; mean ±SD)**	139.51±22.41	142.98±21.03	0.017	144.91±23.21	143.15±21.79	0.345
**DBP (mmHg; mean ±SD)**	86.91±12.27	89.08±12.76	0.014	89.27±12.84	88.03±12.06	0.235
**LDL cholesterol (mmol/L; mean ±SD)**	2.55±0.78	2.85±0.80	<0.001	2.76±0.78	2.67±0.85	0.233
**HDL cholesterol (mmol/L; mean ±SD)**	2.03±0.22	2.08±0.17	0.788	2.04±0.32	2.03±0.19	0.922
**TG (mmol/L; median [IQR])**	1.43±0.88	1.72±1.52	<0.001	1.40±1.05	1.44±0.89	0.594
**TC (mmol/L; median [IQR])**	4.75±0.99	5.08±1.01	<0.001	4.79±0.89	4.77±1.00	0.828
**CRP (mg/L; mean ±SD)**	3.80±6.55	4.60±8.26	0.159	4.72±7.92	4.71±8.65	0.994
**eGFR (ml/min/1.73m2; mean ±SD)**	67.23±20.25	66.62±19.38	0.653	66.60±19.31	66.36±19.60	0.881
**Urine protein positive (%)**	50.96	57.92	0.049	51.44	53.58	0.673

Values for categorical variables are given as a number (percentage); for continuous variables, as mean ± standard deviation.

**§** Normally distributed variables were compared using the Student’s t-test, and non-normal distributed variables were compared using the Mann-Whitney U test. Categorical variables are presented as percentages and were compared using the Chi-square test.

Abbreviations: AF, atrial fibrillation; SBP, systolic BP; DBP, diastolic BP; TG, triglycerides. TC, total cholesterol; CRP, C-reactive protein; LDL, low-density lipoprotein; HDL, high-density lipoprotein; eGFR, estimated glomerular filtration rate.

### Risk of All-cause mortality and stroke in CKD patients with AF

The median follow-up time was 5.56 years (5.69±1.96). During follow-up, there were 70 cases of death, and 16 cases of ischemic stroke and 6 cases of hemorrhagic stroke that occurred among the participants with AF in comparison with 2572 cases of death and 656 cases of ischemic stroke and 184 cases of hemorrhagic stroke among the participants without AF. Before propensity score matching, AF was associated with higher hazard ratios for all-cause mortality (3.97, 95% CI 3.13 to 5.04) and ischemic stroke (2.91, 95% CI 1.77–4.78) and hemorrhagic stroke (3.79, 95% CI 1.68–8.55) compared with that in Non-AF group in unadjusted analyses. After adjusting (Model I = no adjusted; II = adjusted for age, gender; III = adjusted for age, gender, medical history of hypertension and diabetes; IV = adjusted for all covariates except for eGFR and urine protein; V = adjusted for all covariates), the association was slightly affected but observed consistent as before (Table 2).

**Table 2:** Cox proportional hazards analyses of mortality and stroke events among participants with AF before and after propensity score matching.

Model	Pre–Propensity Score Match		Post–Propensity Score Match	
	Hazard Ratio (95% CI)	P Value	Hazard Ratio (95% CI)	P Value
**All-cause Mortality**				
**I**	3.97(3.13–5.04)	<0.001	1.78(1.32–2.40)	<0.001
**II**	2.35(1.85–2.98)	<0.001	1.92(1.42–2.59)	<0.001
**III**	2.37(1.87–3.02)	<0.001	1.92(1.42–2.59)	<0.001
**IV**	2.32(1.78–3.06)	<0.001	1.98(1.44–2.73)	<0.001
**V**	1.86(1.33–2.59)	<0.001	1.51(1.03–2.22)	0.036
**Ischemic stroke**				
**I**	2.91(1.77–4.78)	<0.001	1.61(1.18–2.83)	0.001
**II**	2.35(1.43–3.88)	0.001	1.32(1.10–2.86)	0.010
**III**	2.22(1.33–3.71)	0.002	1.27(1.04–2.76)	0.018
**IV**	2.23(1.26–3.97)	0.006	1.26(1.03–2.77)	0.023
**V**	2.04(1.09–3.83)	0.026	1.20(1.02–2.64)	0.042
**Hemorrhagic stroke**				
**I**	3.79(1.68–8.55)	0.001	2.89(1.29–6.12)	0.005
**II**	3.69(1.63–8.31)	0.002	1.77(1.20–5.88)	0.008
**III**	3.59(1.59–8.09)	0.002	1.60(1.10–5.89)	0.012
**IV**	4.53(2.01–10.23)	<0.001	1.87(1.46–6.00)	0.005
**V**	4.25(1.74–10.36)	0.001	1.65(1.08–5.97)	0.023

I = unadjusted; II = adjusted for age, gender; III = adjusted for age, gender, history of hypertension, diabetes; IV = adjusted for all covariates except for eGFR and urine protein; V = adjusted for all covariates

**Fig 2:**
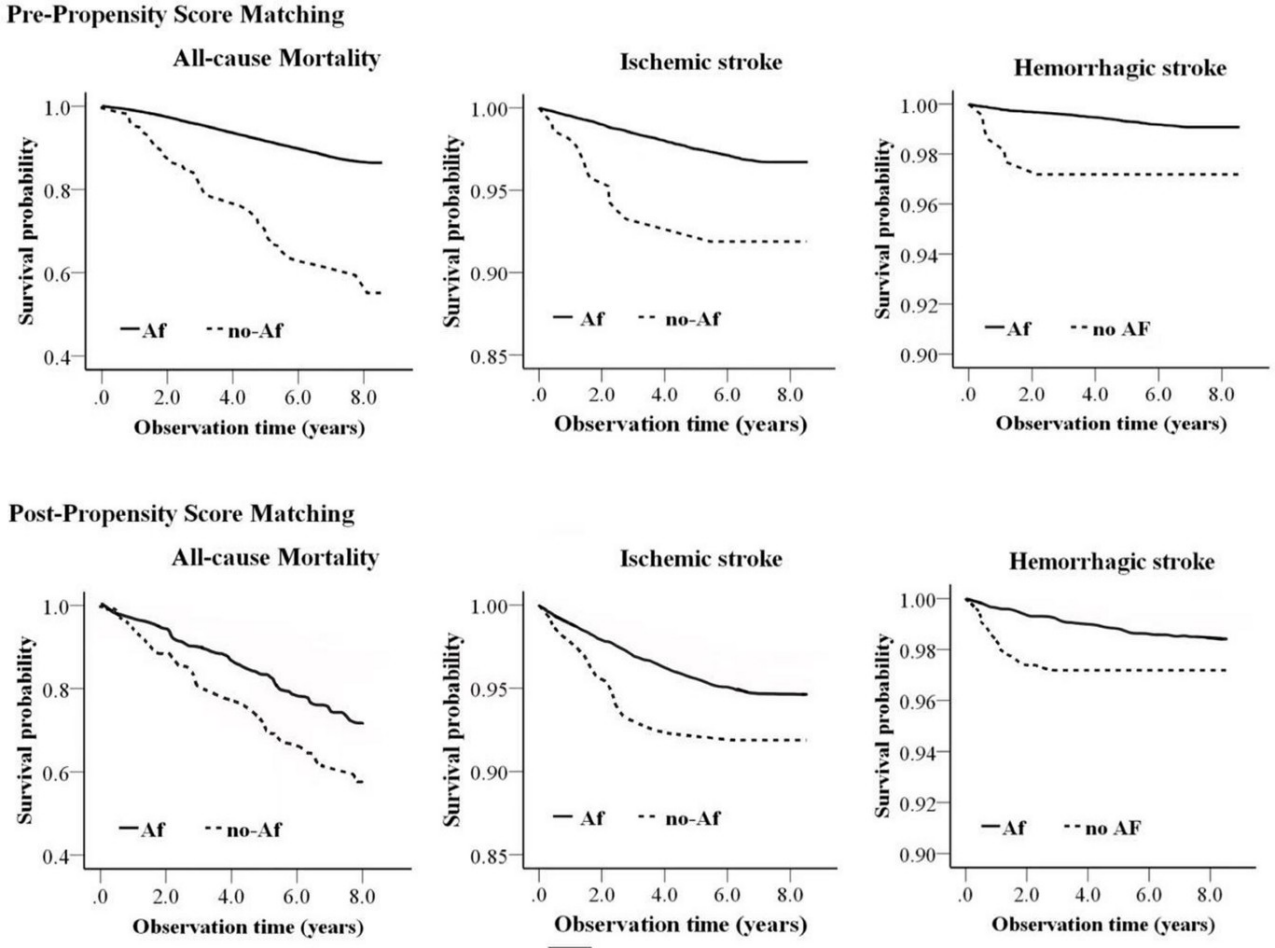
Kaplan-Meier Curve demonstrating the association of AF with all-cause mortality and stroke events among pre- and post-propensity score-matched participants.

After propensity score matching, AF was still significantly associated with higher risks of all-cause mortality, ischemic and hemorrhagic stroke events by uni-variable analyses and multivariable analyses. The difference of all-cause mortality and different types of stroke among Pre- and Post-Propensity Score Matched Participants were also demonstrated by Kaplan-Meier Curves Relating to AF and non-AF group of individuals (Fig 2).

## Discussion

In this community-based longitudinal cohort of Chinese adults with CKD, we found an independent association between AF and increased risks of all-cause mortality and stroke in a subsequent 5-year follow-up, there was an 86% higher risk of all-cause mortality and 104% higher risk of ischemic stroke and 325% higher risk of hemorrhagic stroke in CKD individuals with AF. Furthermore, these associations were still consistent and strong even after strictly propensity score-matched analysis. Although previous literature has shown that AF is an independent risk factor towards these adverse outcomes, most of those studies were performed among western populations, the information from Asian was limited, our data may be beneficial compensatory information in the prevention of AF related adverse outcomes in CKD population.

Previous studies have reported that AF is associated with adverse clinical outcomes in the general population and ESRD patients [17–20]. One of the index studies performed in the general population was from The Framingham Heart Study [21], they examined 5209 general individuals who developed AF and the results showed that AF was associated with a 1.5 fold of increased mortality risk. Another major study was the international Dialysis Outcomes and Practice Patterns Study (DOPPS) which included more than 17000 dialysis patients, AF was also proven to be associated with 28% higher rates of stroke and 16% higher rate of all-cause death [22]. Besides these, the impact of AF towards clinical endpoints in some early stages of CKD also caused attention in recent years. Bansal et al [23] performed an analysis in a group of adults with CKD enrolled in Kaiser Permanente Northern California, among 206229 adults with CKD, 16463 developed incident AF during a 5-year follow-up, the incident AF was proven to be associated with a 67% increase in the rate of ESRD. Sood et al [24] explored the impact of AF towards clinical outcomes like cardiovascular disease and all-cause mortality in patients with a decrease in eGFR, the results showed that Incident AF was associated with a high risk for these adverse outcomes in patients with eGFRs < 90 mL/min per 1.73 m^2^, these difference were more pronounced within the first 6 months of the index date. A recent analysis from the Chronic Renal Insufficiency Cohort (CRIC) Study showed that the incident AF is independently associated with two- to five-fold increased rates of developing subsequent heart failure, myocardial infarction, stroke, or death in adults with CKD [25].

However, all these observational analyses were performed in western populations. In our study based on 21587 Chinese adults with CKD in Kailuan database, we found a higher rate of mortality and stroke among those who developed AF, the AF was associated with 86%, 104% and 325% increase in the rate of death, ischemic stroke and hemorrhagic stroke, respectively. This association was still consistent and strong even after the strict propensity score-matched analysis. Some studies from Asian concerning the pathological role of AF towards outcomes were also performed in general populations. Suzuki et al [26] collected single electrocardiographic AF data from a sample of 13228 Japanese adults in an urban city of Tokyo and found that AF was associated with stroke and all-cause mortality with a nearly 5-year follow-up. Lee et al [27] conducted a prospective cohort study among 3560 Chinese adults in Taiwan, the results indicated that AF was a significant risk factor for stroke and all-cause death in Chinese adults. However, these results were drawn depending on the epidemiological features of AF in several years ago. As the time goes on, the epidemiological features of CKD and AF might have significantly changed which in turn to have a potential influence on the association between AF and adverse outcomes [7], this is also one of the main reasons that we made our new analysis. Altogether, our results extend these earlier observations to a large Chinese population with CKD and provide more supplementary information in understanding of the potential risk of AF existing in CKD adults. To the best of our knowledge, there are no previous studies that have evaluated the relation between AF and adverse outcomes in such a large number of Chinese CKD patients.

Our study had several strengths. We prospectively explored a very large and diverse sample of community-based Chinese CKD adults 45 years and older in a relatively long time of follow up. We also applied the modified Chinese EPI equation to classified CKD on entry into the study cohort to confirm the different stages of CKD. In order to examine the direct and independent influence of AF towards those adverse endpoints, we also applied propensity score analysis to make each of the available variables between AF and non-AF groups matched. Our study also had several limitations. First, we were unable to determine the exact mechanisms explaining the association between AF and clinical outcomes because the study was an observational cohort. Some potential mediators between AF and adverse outcomes like the hormones in the renin-angiotensin-aldosterone system were not examined, the previous episodes of atrial fibrillation were not recorded in our study and some factors like anticoagulation therapy information were also not included in the dataset, as those treatments might influence the incidence of hemorrhagic stroke, it should be paid great attention while applying them in the patients with CKD, these shortages were hopefully added in our future exploration. Second, we were also not able to quantify accurately the severity of proteinuria because only urine dipstick results were available, this might have a negative influence on the identification of CKD individuals in our study. Third, we could not completely rule out residual confounding, although we applied statistically adjustments for a wide range of potential risk factors, like sex, age, blood pressure, and hemoglobin level. Finally, we performed our study among health plan members within a large community-based individual in northeast China, so our findings may not be completely generalizable to other parts of Chinese populations with CKD.

## Conclusion

AF is associated with an 86% higher relative rate of all-cause mortality and 104% that of ischemic stroke and 325% that of hemorrhagic stroke among this group of Chinese adults with CKD, this association is independent of some proved clinical risk factors. Facing the challenge from the combination of these two diseases, future study should focus on elucidating the potential contributing factors which lead to the development of AF in the setting of CKD and potentially modifiable mechanisms through which AF leads to a higher risk of adverse outcomes.

## Supporting information

S1 Table Dataset for basic characteristics and outcomes of the participants in this study. (XLSX)
